# Effect of Chronic Western Diets on Non-Alcoholic Fatty Liver of Male Mice Modifying the PPAR-γ Pathway via miR-27b-5p Regulation

**DOI:** 10.3390/ijms22041822

**Published:** 2021-02-12

**Authors:** Jian Zhang, Catherine A Powell, Matthew K Kay, Ravi Sonkar, Sunitha Meruvu, Mahua Choudhury

**Affiliations:** Pharmaceutical Sciences, Texas A&M Health Science Center, College Station, TX 77843, USA; jianzhang@tamu.edu (J.Z.); c.a.powell29@gmail.com (C.AP.); matt.kay@tamu.edu (M.KK.); rsonkar@uabmc.edu (R.S.); meruvunukala@gmail.com (S.M.)

**Keywords:** high-fat high-fructose diet, PPARγ, noncoding RNA, non-alcoholic fatty liver disease, obesity, diabetes, fibrosis progression

## Abstract

Western diets contribute to metabolic diseases. However, the effects of various diets and epigenetic mechanisms are mostly unknown. Here, six week-old C57BL/6J male and female mice were fed with a low-fat diet (LFD), high-fat diet (HFD), and high-fat high-fructose diet (HFD-HF) for 20 weeks. We determined that HFD-HF or HFD mice experienced significant metabolic dysregulation compared to the LFD. HFD-HF and HFD-fed male mice showed significantly increased body weight, liver size, and fasting glucose levels with downregulated PPARγ, SCD1, and FAS protein expression. In contrast, female mice were less affected by HFD and HFD-HF. As miR-27b contains a seed sequence in PPARγ, it was discovered that these changes are accompanied by male-specific upregulation of miR-27b-5p, which is even more pronounced in the HFD-HF group (*p* < 0.01 vs. LFD) compared to the HFD group (*p* < 0.05 vs. LFD). Other miR-27 subtypes were increased but not significantly. HFD-HF showed insignificant changes in fibrosis markers when compared to LFD. Interestingly, fat ballooning in hepatocytes was increased in HFD-fed mice compared to HFD-HF fed mice, however, the HFD-HF liver showed an increase in the number of small cells. Here, we concluded that chronic Western diet-composition administered for 20 weeks may surpass the non-alcoholic fatty liver (NAFL) stage but may be at an intermediate stage between fatty liver and fibrosis via miR-27b-5p-induced PPARγ downregulation.

## 1. Introduction

The prevalence of Type 2 diabetes (T2D) affecting the modern era is an ongoing crisis pointing to a worldwide epidemic in the upcoming decades [1]. This epidemic is largely based on metabolic syndrome brought about by the worldwide spread of the Western diet, with its high fat content and industry-processed sugars and carbohydrates, causing obesity, insulin resistance, cardiovascular disease (CVD), and metabolic syndrome-related organ dysfunction [2]. One of the more important metabolic syndrome-related diseases is nonalcoholic fatty liver disease (NAFLD), with over one billion individuals estimated to be affected worldwide [3]. NAFLD consists of two subtypes: non-alcoholic fatty liver (NAFL), a fatty liver without inflammation which is generally considered to be benign, and nonalcoholic steatohepatitis (NASH) [4,5]. Generally, it is believed that only NASH patients have potential for developing fibrosis progression and cirrhosis development [6]. Though previous studies have suggested that NAFL may be benign, and does not lead to progressive fibrosis [4], emerging data suggest that fibrosis progression may be seen not only in NASH but also in NAFL [5,7,8]. As such, most individuals with NAFLD may be at risk for advanced liver injury without early intervention.

Overconsumption of a high-fat diet (HFD) and increased intake of sweetened beverages are major risk factors for NAFLD [9]. There is a direct link between fructose consumption and long-term liver damage from NAFLD [10,11]. Additionally, increased fructose intake has been linked with progression of fatty liver tissue to more advanced disease stages, including fibrosis, while decreased fructose intake leads to improvement in NAFLD [12,13,14]. In our study, we have compared the effects of a low-fat diet (LFD) with a high-fat diet (HFD) and a high fat-high fructose diet (HFD-HF) on the liver.

Currently, understanding of hepatic fatty liver is moving into new frontiers. The limitations in preventive measures and lack of established therapies for chronic liver diseases highlight the urgent need to develop novel preventive therapeutic strategies for halting this progression. It has been established that there is a vast complexity in the transcriptional control pathways in the diseased liver [15]. These molecular discoveries provide potential drug targets for intervention. Transcription factor peroxisome proliferator-activated receptor-γ (PPARγ) is one of these attractive targets.

PPARγ is most highly expressed in adipose tissue, where it serves an essential role in the regulation of adipocyte differentiation, adipogenesis, and lipid metabolism [16]. However, it is also an important molecule for adjusting the liver’s response to a HFD and directing its overall regulation for processing free fatty acids, as well as liver regeneration and activation of quiescent hepatic stellate cells (HSCs) [17,18]. In fact, PPARγ deletion in mouse hepatocytes has been shown to be protective against development of steatosis [19,20,21], but this also exacerbates insulin resistance [20,22]. There are many studies investigating the effects of an HFD on the overall metabolism and PPARγ regulation pathways in various metabolic tissues. However, many of these studies were performed solely with HFD compared to chow diet. Virtually no animal studies have been carried out comparing an LFD versus HFD and HFD-HF or within the context of epigenetic regulation.

Epigenetic modulators such as DNA methylation, histone modification, and non-coding RNAs, including microRNAs (miRNAs), stimulate molecular effects on gene and protein expression levels [23,24]. miRNAs work by repressing target gene expression at the post-transcriptional level and many can target nuclear receptors and other genes encoding chromatin altering proteins [25,26]. Recently, evidence showed that NAFLD-related gene transcription affected by miRNAs (or miRs) involves PPARγ, which contains a seed sequence specific for miRs [27,28].

To our knowledge, no study has investigated the effects of three different Western diets exposure on PPARγ-microRNA regulation in the liver. Therefore, this study investigates the effects of Western diets on metabolic syndrome and microRNA regulation of PPARγ.

## 2. Results

### 2.1. Effect of Western Diets on Body Weight and Food Consumption

**Figure 1:**
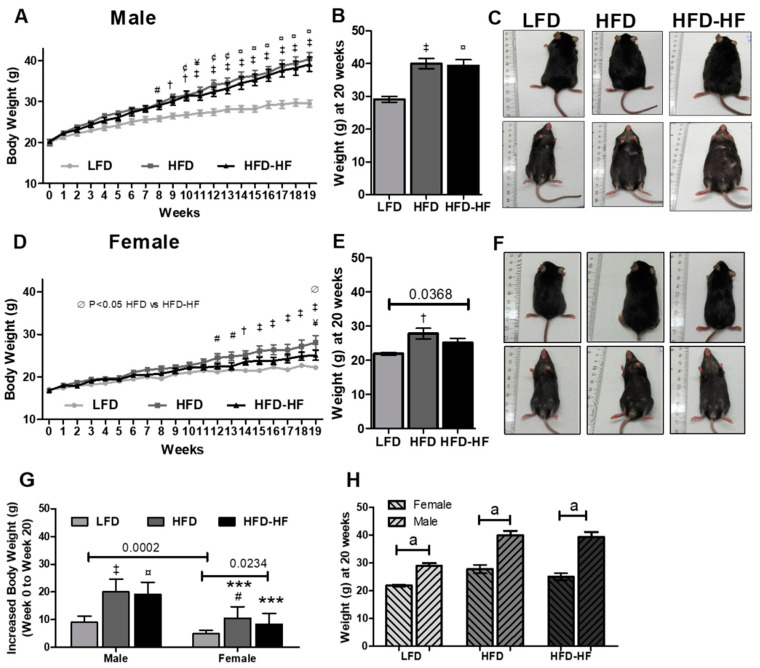
High fat and high fat-high fructose diets increase body weight in male and female mice. Body weight of C57BL/6J male (**A**) and female (**D**) mice placed on LFD (Low-fat diet) (light grey circles), HFD (High-fat diet) (dark grey squares), or HFD-HF (High-fat high-fructose diet) (black triangles) for 19 weeks was measured weekly. (**B,E**) Body weight of LFD (grey bar), HFD (dark grey bar), and HFD-HF (black bar) mice on a diet for 20 weeks. n = 10 mice/group, two-way repeated measures ANOVA for A&D, *t*-test and one-way ANOVA for B&E, ^#^ *p* < 0.05 LFD vs. HFD, ^†^ *p* < 0.01 LFD vs. HFD, ^‡^ *p* < 0.001 LFD vs. HFD, ^¥^ *p* < 0.05 LFD vs. HFD-HF, ^¢^ *p* < 0.01 LFD vs. HFD-HF, ^¤^ *p* < 0.001 LFD vs. HFD-HF, ^∅^ *p* < 0.05 HFD vs. HFD-HF. Representative images of gross mouse phenotype of male (**C**) and female (**F**) mice fed LFD (left panels–rear and front), HFD (middle panels– rear and front), or HFD-HF (right panels–rear and front) for 20 weeks. (**G**) Increased body weight between 0 and 20 weeks in male and female mice. n = 9–10, *t*-test and one-way ANOVA, ^‡^ *p* < 0.001 LFD vs. HFD, ^¤^ *p* < 0.001 LFD vs. HFD-HF, ^#^ *p* < 0.05 LFD vs. HFD, *** *p* < 0.001 Female vs. Male. *p* value of *t*-test was shown. (H) Body weights between male and female mice in the same diet group. n = 9–10 mice/group, *t*-test was performed, ^a^ *p* < 0.0001.

To determine the effect of Western diets on liver, we fed six-week-old male and female mice LFD, HFD, and HFD-HF and measured body weight weekly for 20 weeks. Male mice on HFD and HFD-HF weighed significantly more than LFD-fed mice starting at 8 weeks for HFD (*p* < 0.05) and 10 weeks for HFD-HF (*p* < 0.01) (Figure 1A). HFD and HFD-HF mice weighed significantly more than LFD mice at the 20 week endpoint (Figure 1B,C). Increased body weight (between 0 and 20 weeks) was significantly higher in HFD (*p* < 0.001) and HFD-HF (*p* < 0.001) mice compared to that of LFD mice (Figure 1G, left side). Female mice fed HFD and HFD-HF weighed significantly more than LFD-fed mice starting at 12 weeks for HFD (*p* < 0.05) and week 19 for HFD-HF (*p* < 0.05). Additionally, at week 19, HFD weighed significantly more than HFD-HF mice (*P* < 0.05) (Figure 1D). HFD (*p* < 0.05) and HFD-HF (*p* = 0.0368, *t*-test) mice weighed significantly more than LFD mice at the 20 week endpoint (Figure 1E,F). However, food consumption remained similar in LFD, HFD, and HFD-HF cages over the duration of the experiment for both male and female mice (Supplementary Figure S1). Female HFD (*p* < 0.05) and HFD-HF (*p* = 0.0234, *t*-test) mice showed significantly higher increased body weight than that of LFD mice (Figure 1G Right). Male mice gained significantly more body weight than female mice (*p* = 0.0002, *t*-test for LFD; *p* < 0.001, one-way ANOVA for HFD and HFD-HF, respectively, Figure 1G). Male mice had significantly higher body weight than the female mice in same diet group (Figure 1H, ^a^ *p* < 0.0001, *t*-test). HFD and HFD-HF increase white adipose tissue and subcutaneous adipose tissue weight in male and female mice (Supplementary Figure S2).

### 2.2. Altered Fasting Blood Glucose on Western Diet

**Figure 2:**
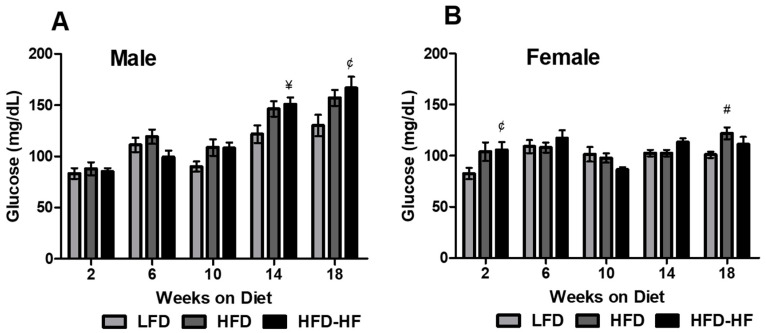
High fat high fructose diet exacerbates diabetic phenotype in male mice (**A**) but not in female mice (**B**). Fasting blood glucose was measured in LFD (light grey bars), HFD (dark grey bars), and HFD-HF (black bars) mice at 2, 6, 10, 14, and 18 weeks on diet. n = 8 mice/group, repeated measures two-way ANOVA, ^#^ *p* < 0.05 LFD vs. HFD, ^¥^ *p* < 0.05 LFD vs. HFD-HF, ^¢^ *p* < 0.01 LFD vs. HFD-HF.

Fasting blood glucose was measured every 4 weeks starting at 2 weeks to 18 weeks to determine the diabetic onset. Male HFD-HF mice exhibited significantly increased fasting blood glucose at 14 weeks (*p* < 0.05) and 18 weeks (*p* < 0.01) compared to LFD (Figure 2A). HFD-HF mice had an average fasting blood glucose of above 150 (mg/dL) (the diabetic cutoff for diabetes) in mice at 18 weeks (Figure 2A). HFD-fed mice did not have significantly elevated fasting blood glucose compared to LFD, nor did the fasting blood glucose levels reach diabetic levels at 18 weeks (Figure 2A). For female mice, only HFD mice exhibited significantly increased fasting blood glucose at 18 weeks (*p* < 0.05) compared to LFD mice (Figure 2B). Interestingly, HFD-HF mice showed significantly increased blood glucose only as early as 2 weeks (*p* < 0.01) but not at other time points compared to LFD mice (Figure 2B). Furthermore, the fasting blood glucose levels did not reach diabetic levels in female mice (Figure 2B).

### 2.3. Increased Liver Weight under Western Diet Exposure

**Figure 3:**
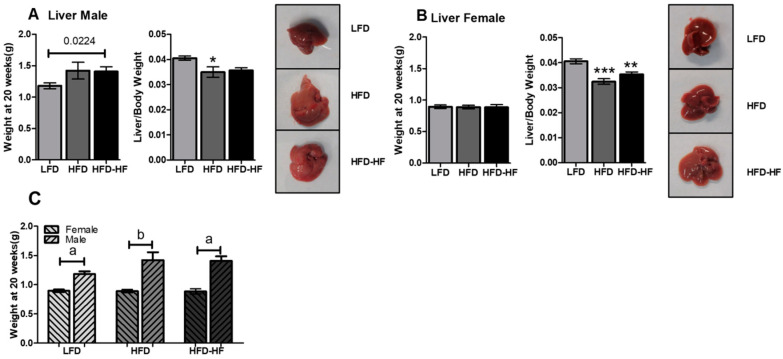
High fat and high fructose diets alter liver phenotype in male mice but not in female mice. Liver weights and ratio of liver and body weight of male (**A**) or female (**B**) mice fed LFD (grey bar), HFD (dark grey bar), and HFD-HF (black bar) with representative images of livers ex vivo from mice fed LFD (upper panel), HFD (middle panel), and HFD-HF (lower panel) for 20 weeks. n = 9–10 mice/group, one-way ANOVA with Tukey’s post hoc test and *t*-test, * *p* < 0.05, ** *p* < 0.01, *** *p* < 0.001. (**C**) Liver weights between male and female mice in the same diet group. n = 9–10 mice/group, *p* value of *t*-test was shown, ^a^ *p* < 0.0001, ^b^ *p* = 0.0010.

The liver weights were increased but not significantly from mice fed HFD or HFD-HF compared to LFD for males (Figure 3A) when compared using one-way ANOVA. Liver weight in HFD-HF mice was significantly higher (*p* = 0.0224) than that of LFD mice in males when compared using *t*-test analysis. The average liver weights in male mice were 1.181 g for LFD, 1.421 g for HFD, and 1.408 g for HFD-HF, respectively. However, the ratio of liver and body weight was significantly lower in HFD but not in HFD-HF when compared to LFD. No significant difference in liver weights was found among three groups of mice for females (Figure 3B). However, the liver to body weight ratio was significantly lower in HFD and HFD-HF compared to LFD. The average liver weight of the 3 groups in females were 0.8946 g (LFD), 0.8890 g (HFD), and 0.8846 g (HFD-HF), respectively. Male mice had significantly higher liver weight than the female mice in same diet group (Figure 3C, *p* < 0.001, *t*-test).

### 2.4. Altered Glucose Tolerance and Insulin Sensitivity under Western Diets Exposure

**Figure 4:**
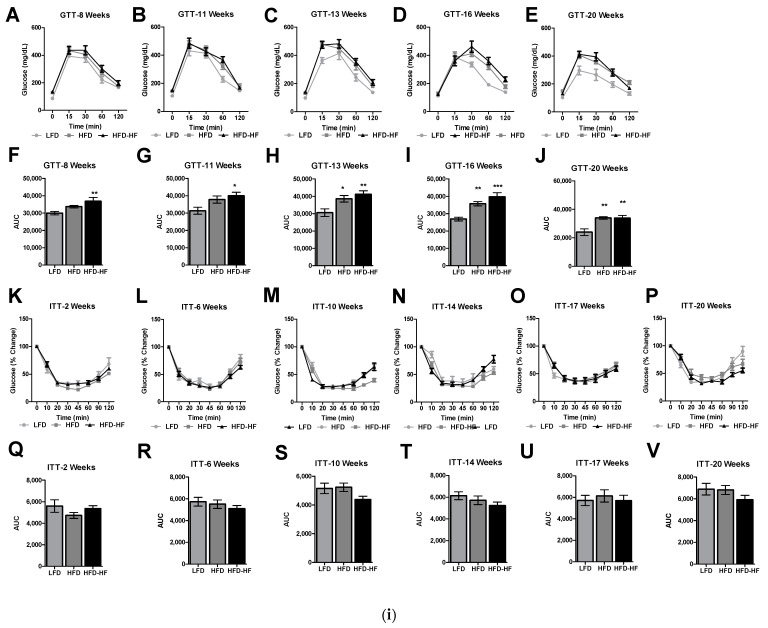

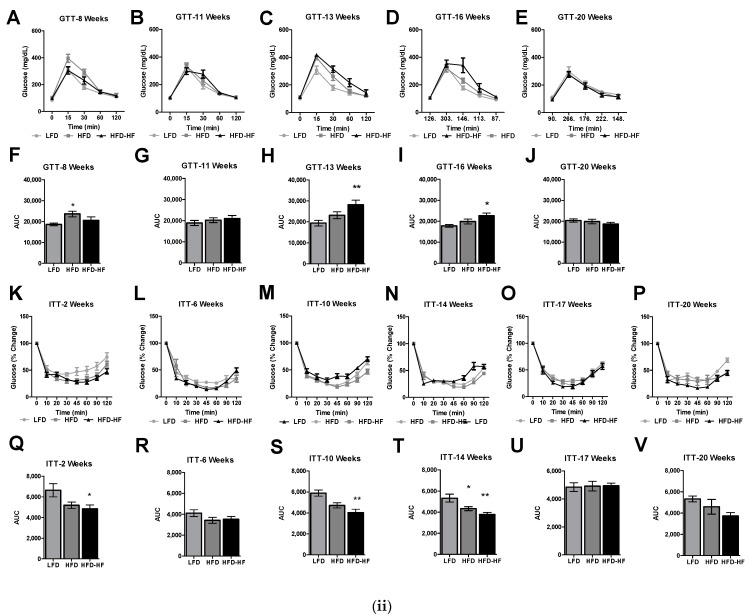
Fructose leads to early perturbations in glucose intolerance in HFD-HF fed male mice (**i**) but not in female mice (**ii**) GTTs were performed at (**A**) 8 weeks, (**B**) 11 weeks, (**C**) 13 weeks, (**D**) 16 weeks, and (**E**) 20 weeks on LFD (light gray, circle), HFD (dark gray, square), or HFD-HF (black, triangle) fed male and female mice. (**F**–**J**) Area under the curve analysis of corresponding panels (**A**,**E**) (Light grey bars-LFD, dark grey bars-HFD, black bars-HFD-HF). ITTs were performed as (**K**) 2 weeks, (**L**) 6 weeks, (**M**) 10 weeks, (**N**) 14 weeks, (**O**) 17 weeks, and (**P**) 20 weeks on LFD (light gray, circle), HFD (dark gray, square), or HFD-HF (black, triangle) fed male and female mice. (**Q**,**V**) Corresponding area under the curve analysis of ITTs performed in panels **K**–**P** (Light grey bars-LFD, dark grey bars-HFD, black bars-HFD-HF). n = 7–8 mice/group, one-way ANOVA with Tukey’s post hoc test, * *p* < 0.05, ** *p* < 0.01 and *** *p* < 0.001 compared to LFD.

Diabetic phenotype was analyzed by assessing glucose tolerance and insulin tolerance for 20 weeks. For males, HFD-HF mice showed early perturbations of glucose intolerance at 8 weeks by GTT compared to LFD (*p* < 0.01), where HFD had no significant change in glucose clearance (Figure 4iA&F). HFD-HF mice and HFD mice had impaired glucose clearance compared to LFD from 13 weeks to 20 weeks (*p* < 0.01) (Figure 4iC–E; H–J). However, HFD-HF did show an increased trend with impaired glucose clearance compared to HFD from 11 weeks to 16 weeks (Figure 4iG–I). There were no changes in ITT as insulin tolerance was not significantly altered by HFD or HFD-HF compared to LFD from 2 weeks to 20 weeks (Figure 4iK–V). For females, HFD mice showed significantly slower blood glucose clearance at 8 weeks by GTT compared to LFD (*p* < 0.05), whereas HFD-HF had no significant change in glucose clearance compared to LFD, or HFD mice (Figure 4iiA&F). HFD-HF mice had impaired glucose clearance compared to LFD at subsequent time points at 13 and 16 weeks (*p* < 0.01) (Figure 4iiC,D,H&I). HFD-HF did not exacerbate impaired glucose clearance compared to HFD (Figure 4iiA–J). However, HFD-HF showed significant insulin sensitivity over LFD at 2, 10 and 14 weeks (*p* < 0.05, 0.01, 0.01, respectively) (Figure 4iiK&Q, M&S, N&T) by ITT. Insulin sensitivity was also significant in HFD mice compared to that of LFD mice at 14 weeks (*p* < 0.05) (Figure 4iiN,T).

### 2.5. Altered Liver Regulatory Markers under Western Diets Exposure

**Figure 5:**
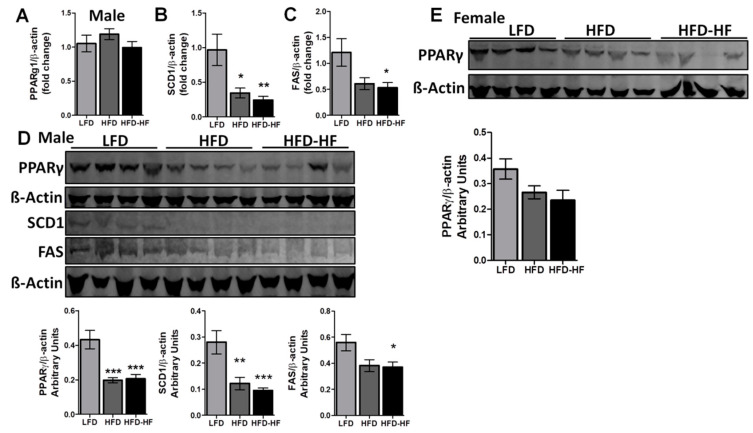
PPARγ downregulation in the livers of HFD and HFD-HF fed male mice but not female mice. Livers of LFD, HFD, and HFD-HF mice on diet for 20 weeks were analyzed for liver regulatory markers. Gene expression of (**A**) PPARγ, (**B**) SCD1 and (**C**) FAS in male livers were analyzed, with expression normalized to ß-Actin. (**D**) Protein expression of PPARγ, SCD1, and FAS were analyzed in livers of LFD, HFD, and HFD-HF male mice by Western Blot. ß-Actin was used as a loading control. Densitometry analysis was performed in male mice (**D**) bar graph under blot PPARγ (left) SCD1 (middle), and FAS (right). (**E**) Protein expression of PPARγ and PPARγ densitometry in liver of female mice. n = 8 mice/group, one-way ANOVA with Tukey’s post hoc test, * *p* < 0.05, ** *p* < 0.01, *** *p* < 0.001 compared to LFD.

Hepatic PPARγ expression is robustly induced in NAFLD patients and experimental models [22]. In our study, PPARγ gene expression was not altered in the mouse livers among the three diets (Figure 5A). However, gene expression of SCD1 was significantly decreased in HFD (*p* < 0.05) and HFD-HF (*p* < 0.01) mice compared to LFD mice (Figure 5B), and gene expression of FAS was significantly decreased in HFD-HF (*p* < 0.05) mice compared to LFD mice (Figure 5C). To confirm this trend, protein expression of PPARγ, SCD1, and FAS were analyzed in livers of LFD, HFD, and HFD-HF male mice (Figure 5D). Interestingly, protein expression of PPARγ was significantly decreased in HFD (*p* < 0.001) and HFD-HF (*p* < 0.001) mice compared to LFD mice, only for males (Figure 5D) but not for females (Figure 5E).

As the female mice did not show any changes in PPARγ protein expression, we examined only the male mice groups afterward. Protein expression of SCD1 (Figure 5D) was significantly decreased in HFD (*p* < 0.01) and HFD-HF (*p* < 0.001), however, protein expression of FAS (Figure 5D) was only significantly decreased in HFD-HF (*p* < 0.05) compared to male LFD mice.

### 2.6. Altered MicroRNA-27 Expression under Western Diets Exposure

**Figure 6:**
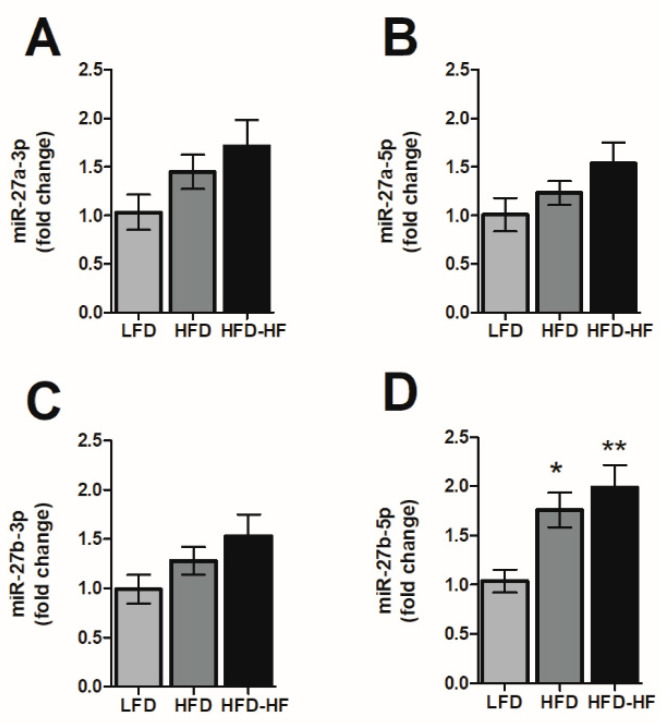
microRNA expression changes in male liver**.** miRNA expression was analyzed in the livers of male mice on LFD, HFD, or HFD-HF for 20 weeks. Expression changes of (**A**,**B**) miR-27a-3p/-5p and (**C**,**D**) miR-27b-3p/-5p in male liver between the LFD, HFD, and HFD-HF groups. n = 7 mice/group, one-way ANOVA with Tukey’s post hoc test, * *p* < 0.05, ** *p* < 0.01 compared to LFD.

Recent evidence reveals several scenarios of miRNAs accumulating in hepatocytes by NAFLD dysregulation. miRNAs have been shown to possess the ability to directly bind proteins [29] and induce gene expression as well [30]. miRNAs are known to have a direct impact on the expression of PPARγ, which mediates expression of many downstream genes [18]. In particular, miR-27a and miR-27b have been shown to have a direct effect in downregulating PPARγ expression [31,32,33]. However, the microRNA subtypes regulation under diets are not known. In our study, expression of miR-27b-5p miRNA was significantly increased in livers of male HFD (*p* < 0.05) and HFD-HF (*p* < 0.01) mice compared to LFD (Figure 6D). However, even though expression of miR-27a-3/5p and miR-27b-3p were increased from LFD to HFD and HFD-HF, the levels were not significant between diets (Figure 6A–C).

### 2.7. Fibrosis Markers under Western Diets Exposure

**Figure 7:**
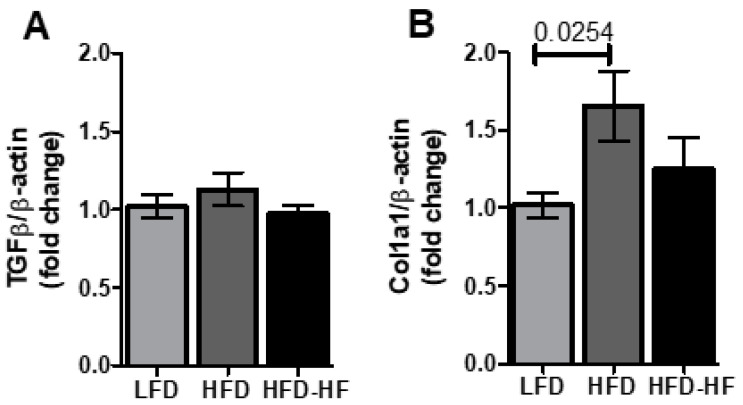
mRNA expression of fibrosis regulators in male liver. Expression changes of (**A**) TGFβ and (**B**) Col1a1 in liver among the LFD, HFD, and HFD-HF groups for 20 weeks. n = 7 mice/group, *t*-test.

In order to investigate whether Western diets modulate fibrosis markers, we measured three well-known fibrosis biomarkers. TGFβ was not changed by HFD and HFD-HF. (Figure 7A). In the case of Col1a, HFD increased hepatic gene expression of Col1a1 (*p* = 0.0254 in a *t*-test) compared to LFD; however, the HFD-HF did not show any significant changes when compared to LFD (Figure 7B). Furthermore, even though a HFD showed a significant increase in PAI-1 gene expression, the Ct value of PAI-1 was around 30 and above in all samples for three groups, indicating a very minimal expression of this gene, which further indicates that full blown fibrosis has not yet been developed (Supplementary Figure S3). The Ct value for other genes are between 18–25. In all cases, we carried out *t*-test analysis as one-way ANOVA did not show any significance (Figure 7B).

### 2.8. Lipid Accumulation under Western Diets

**Figure 8:**
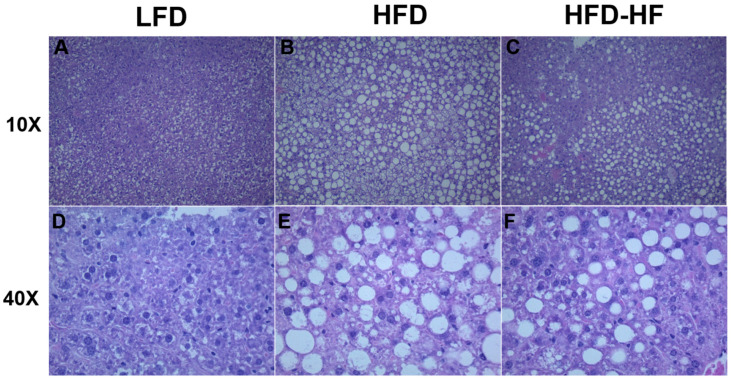
Lipid accumulation under Western diets. Representative Brightfield photomicrographs of 10× and 40× magnification H&E stained liver sections of male mice on LFD, HFD, or HFD-HF for 20 weeks. (**A**,**D**) Image corresponds to LFD mouse liver, with normal lobular structure, without signs of anomaly, and smaller cell size. (**B**,**E**) Image corresponds to HFD mouse liver, with large deposits of lipids present and much larger cells present. (**C**,**F**) Image corresponds to HFD-HF mouse liver, with less frequent large deposits of lipids compared to the HFD image, but with an increase in the number of smaller cells present.

H&E stained liver images show normal lobular structure without signs of anomaly in LFD male mice (Figure 8A,B). In contrast, large deposits of lipids are found in HFD mouse livers (Figure 8B,E). Interestingly, the image corresponding to HFD-HF mouse liver (Figure 8C,F) shows less frequent large lipid deposits compared to the HFD mice (Figure 8B,E). We also observe a corresponding increase in number of small cells in HFD-HF liver (Figure 8C,F).

## 3. Discussion

Identification of therapeutic targets to treat or prevent NAFLD has been limited despite extensive efforts, treatments, and preventive interventions. While there is certainly a genetic component to these complex diseases, the recent increase in liver abnormalities cannot be caused by genetic changes alone in the population [34]. Recently, the interaction between genes and the diet has emerged as a new frontier for the discovery of how networks of modified genes contribute to several major pathologies [35]. The current evidence is still incomplete. Few of the aspects are due to a lack of comparison of diets in attaining NAFLD or the complex steps of NAFLD or whether any epigenetic regulatory pathway controls the well-known genetic modulators under these diets, and therefore more efforts are called for to accurately describe the progression during NAFLD (e.g., from healthy to NAFL to NASH). Here, we compared three different diets’ effect on well-known liver regulators and provide evidence of a potential microRNA regulation leading to an advanced stage of NAFLD in a mouse model.

We observed that the male mice showed significant metabolic phenotype abnormalities compared to female mice under different Western diets (Figure 1, Figure 2, Figure 3 and Figure 4). The primary for the sex-specific result is that the female C57BL/6J mouse is well known for responding poorly to the obesogenic diet compared to male C57BL/6J mice, which is known to be a well-studied obesogenic model. These different responses to the HFD and HFD-HF challenges are also due to different hormonal responses which have been previously documented [36,37,38]. These sex difference factors bear merit for future investigations, possibly in a different model, specifically primate [39]. We observed that there were increased glucose levels in the glucose tolerance test, yet no significant insulin sensitivity differences in HFD-HF groups. Therefore, this evidence supports the fact that higher glucose levels precede insulin resistance or diabetes. A fully manifested picture of diabesity in mice has been shown to develop after 16–18 weeks of high-calorie diet regimen when compared to chow diet [40]. Here, we have to consider that the control group in this study is an LFD, not a chow diet. The chow diet is, in general, the control diet for most diabetes and obesity research. However, healthy humans do consume some amount of fat in day-to-day life. Therefore, we decided to compare HFDs with an LFD to simulate a human diet more realistically. Furthermore, use of a LFD for diabetes-obesity studies is also strongly suggested rather than a chow diet, which may help the NIH mission for more rigor and reproducibility [41,42]. A previous study supports the fact that when comparing a LFD with 45% HFD, significant insulin resistance was not observed, but did show up when a higher percentage of fat diet was used in another group [43]. Furthermore, in individuals without diabetes, the addition of fructose to diets in isocaloric exchange for other macronutrients does not affect insulin sensitivity [44,45]. Therefore, we conclude that long-term high calorie diets would eventually lead to insulin resistance.

At the molecular level, we then investigated PPARγ regulation in liver under three different fat containing diets (Figure 5). Interestingly, PPARγ protein expression was significantly downregulated to a similar level in HFD and HFD-HF fed mice compared to LFD. As evidenced before, hepatic expression of PPARγ is increased in people with NAFL [46] while decreased during liver fibrogenesis in NASH [47,48]. Zhang et al. has an interesting finding where downregulation of FAS and SCD1 had been shown in high carbohydrate and high fat diets but did not have the same effect on PPARγ [49]. Therefore, we investigated the downstream pathway regulators FAS and SCD1, which showed reduced expression. A study involving PPARγ transgene liver-knockout mice also showed the similar decrease in FAS and SCD1 [50]. Reduction of these enzymes in fatty acid pathways leads to an increase in triglyceride assembly and export. Eventually, triglyceride accumulation overcomes the liver’s ability to export it, leading to excess lipid storage and ballooning of hepatocytes, symptoms of NAFL.

**Figure 9:**
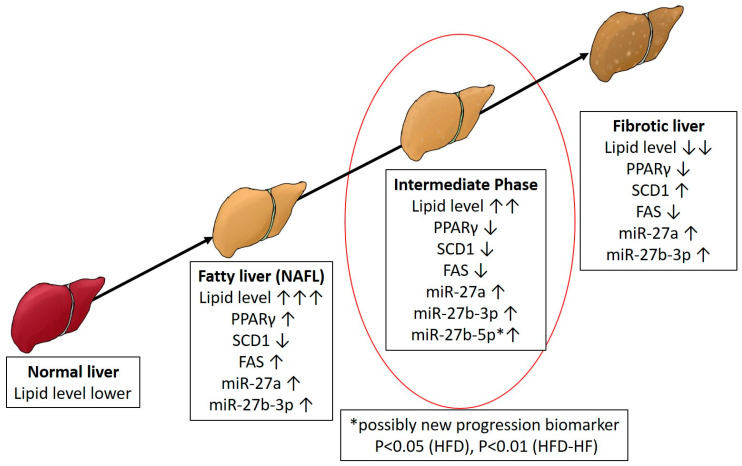
Proposed Model of Western diet induced intermediate stage between fatty liver and fibrosis via epigenetic regulation. Epigenetic and genetic molecular pathways are shown to be regulated by high fat high fructose diet. The diet induces an intermediate state of liver disease progression where molecular signaling indicates of fibrosis progression in NAFL but no phenotypic appearance yet. Proposed potential microRNA biomarkers are shown during the progression along with transcriptional changes. * Possibly new progression biomarker *p* < 0.05 (HFD), *p* < 0.01 (HFD-HF).

Previous evidence typically demonstrated that FAS and SCD1, PPARγ in an inverse correlation between fatty liver and fibrosis (Figure 9) [46,47,48,49,50,51,52,53,54,55,56]. As our findings do not show the typical hallmarks of fatty liver, we investigated several fibrosis markers. Fibrosis markers investigated here showed insignificant changes when compared between diets or minimal expression (Figure 7, Supplementary Figure S3). As molecular events surrounding PPARγ under our experimental diets show a combination of characteristics of fatty liver and fibrosis, we conclude that our experimental model may be initiating a molecular step toward fibrosis progression in the NAFL stage, but has not yet reached the clinical fibrosis stage at the genetic level (Red circle, Figure 9). Indeed, this is possible as NAFLD patients without steatohepatitis have been shown to develop progressive fibrosis [5]. Therefore, investigations at the epigenetic level are significant.

Recent evidence has shown that NAFLD-related gene transcription affected by miRs involves PPARγ, which contains seed sequences for miRs. Ample evidence shows a potential role of miR-27 in NAFLD, specifically in the context of PPARγ as it has a direct seed sequence. Our study’s dietary regimen specifically regulated a significant increase in miR-27b-5p (Figure 6D), but not the other miR-27 subtypes. MiR-27b has also been proposed in a panel for high diagnostic accuracy in identifying NAFLD [57] and has also been elevated in NASH livers of rats and zebrafish [58]. Povero et al. showed that extracellular vesicles (EV) released by hepatocytes during lipotoxicity can carry and transfer miRNAs that can regulate fibrogenesis [20]. These EVs are released during cell stress or death and are key cell-to-cell communicators, which can be internalized by neighboring cells and initiate hepatocyte dysregulation [21]. Their study confirmed the presence of miR-27b within EVs isolated from fat-laden hepatocytes, their transmission into non-fatty quiescent HSC, and the subsequent downregulation effects on PPARγ expression and activation/proliferation of HSC leading to fibrosis [20]. Observing that PPARγ downregulation leads to the promotion of liver fibrosis in NAFLD [18], we could potentially tie in the additional increase in smaller fat droplets seen in the liver (Figure 8C,F), specifically the increase in the fraction of smaller cells visible, with an increase in miR-27-5p.

**Figure 10:**
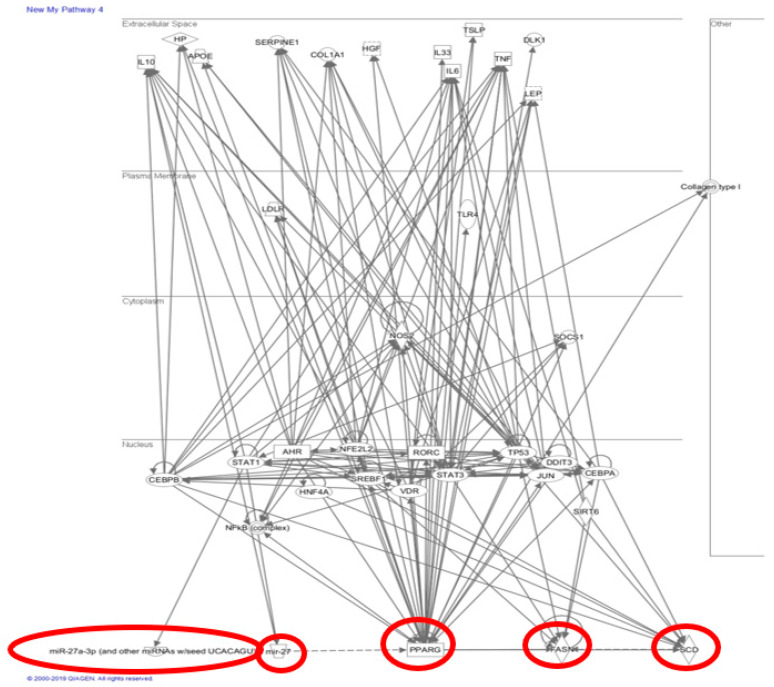
Ingenuity Pathway Analysis. Markers of interest (PPARγ, miR-27, SCD1, FAS) were entered into the IPA program by Invitrogen to search for relevant connections between regulatory pathways. Unrelated downstream markers are removed; only direct connections are shown.

Our study proposes an “intermediate liver stage” during NAFLD where fibrosis may have been initiated at the NAFL stage under the influence of a Western diet (red circle, Figure 9). Here, we conclude that diet may induce interactions at the molecular epigenetic level, which progresses to surpass fatty liver (red circles, Figure 9 and Figure 10). Therefore, our study provides an insight that having an intermediate stage with a potential epigenetic biomarker can help to prevent or reverse the progression of adverse liver anomalies. Future studies will generate miR-27-subtypes specific knockout mice and investigate the effects of different Western diets to observe whether downregulating specific miR-27 subtypes can be protective to liver abnormalities.

## 4. Material and Methods

### 4.1. Mice

Five-week-old C57Bl/6 male and female mice were purchased from the Jackson Laboratory. Mice were acclimated for one week before the start of the study. Mice were fed chow diet (PicoLab Rodent Diet 5053, Lab Supply, Fort Worth, TX, USA) and water ad libitum in 12:12 h light/dark controlled room for one week.

Six-week-old mice were then fed low-fat diet (LFD, 10% energy from fat), high-fat diet (HFD, 45% energy from fat), or high-fat high-fructose (HFD-HF, 45% energy from fat, 17% energy from fructose) (Research Diet D12450H, D12451Y, D15041701) for 20 weeks [59] (Supplementary Table S1). All diets were balanced to have equal amounts of kilocalories available (4057 kcal per weight) (Research Diets, Inc. New Brunswick, NJ, USA). Body weight was measured weekly. At the end of the study, mice were fasted for 6h and sacrificed under anesthesia. Liver was excised, weighed, and flash frozen in liquid nitrogen or fixed in 10% buffered formalin prior to paraffin embedding. All procedures were approved by Texas A&M IBT IACUC (IACUC 2014-0338-IBT).

### 4.2. Body Weight and Fasting Blood Glucose

Non-fasted mice were weighed weekly (same day of the week and time, e.g., ~9:00 am.). Mice were fasted overnight and fasting blood glucose was measured within 14–16 h of fasting using a glucometer (McKesson). A blood sample was drawn from the tail and applied to the glucose test strip. The first blood sample (drop) was discarded and measurements were performed on the second blood sample.

### 4.3. Glucose and Insulin Tolerance Tests

Animals were fasted overnight and a glucose tolerance test (GTT) was performed using 2 g of glucose (Sigma, St. Louis, MO, USA) per kilogram body weight, administered by intraperitoneal injection. Glucose readings were taken at baseline (time = 0 min) and at 15, 30, 60, and 120 min at tail vein after injection.

An insulin tolerance test (ITT) was conducted using insulin (Sigma, St. Louis, MO, USA) at 0.75 Units/kg body weight (male) and 0.6 Units/kg body weight (female) administered by intraperitoneal injection. Animals were fasted (5 h), and blood glucose was tested by tail vein at baseline (time = 0 min) and at 10, 20, 30, 45, 60, 90, and 120 min after injection.

### 4.4. miRNA Real Time PCR

miRNA was extracted using E.Z.N.A. miRNA Kit (Omega Bio-tek, Norcross, GA, USA) according to the manufacturer’s protocol as previously described [60]. cDNA synthesis and miR-specific quantitative real-time PCR was performed. miRNA expression was normalized to U6 and expressed as relative expression compared to control low fat diet. Relative miRNA expression was analyzed using 2 ^−ΔΔCt^ method and represented as fold change [60]. The primer sequences were: U6 F-5′-CGCAAGGATGACACGCAAATTC-3′, U6 R-5′-AGGTCCAGTTTTTTTTTTTTTTTAAAATATG-3′; miR-27a-3p F-5′-GCAGTTCACAGTGGCTAAG-3′, R-5′- CCAGTTTTTTTTTTTTTTTGCGGA-3′; miR-27a-5p F-5′-AGGGCTTAGCTGCTTGT, R-5′-TCCAGTTTTTTTTTTTTTTTGCTCA-3′; miR-27b-3p F-5′- GCAGTTCACAGTGGCTAAG-3′, R-5′- TCCAGTTTTTTTTTTTTTTTGCAGA-3′; miR-27b-5p F-5′-GAGAGCTTAGCTGATTGGTG-3′, R-5′-GGTCCAGTTTTTTTTTTTTTTTGTTC-3′.

### 4.5. mRNA Real Time RT-PCR

mRNA was extracted according to E.Z.N.A. miRNA kit according to the manufacturer’s protocol (Omega Bio-tek, Norcross, GA, USA). Reverse transcription (RT) was carried out using the High-Capacity cDNA Reverse Transcription Kit according to the manufacturer’s protocol (Life Technologies, Carlsbad, CA, USA). One microgram of mRNA was reverse transcribed to cDNA according to the manufacturer’s instructions. The expression levels of gene transcripts were determined using quantitative real-time PCR. Primers for the PCR were designed to span exon/exon junctions to minimize amplification of residual genomic DNA using primer blast. The primer sequences were: β-actin F- 5′-AGCCATGTACGTAGCCATCC-3′, R-5′- GCTGTGGTGGTGAAGCTGTA -3′; SCD1 F-5′- GTCAAAGAGAAGGGCGGAAAAC-3′, R-5′- AAGGTGTGGTGGTAGTTGTGGAAG-3′; FAS F-5′- GCTGCGGAAACTTCAGGAAAT-3′, R-5′- AGAGACGTGTCACTCCTGGACTT-3′; PPARgamma1 F-5′- AGAAGCGGTGAACCACTGATATTC-3′, R-5′- AGAGGTCCACAGAGCTGATTCC-3′; TGFβ F-5′- TGCAGATTGTCAAGGAAGTGTC -3′, R-5′- AGGCCAGGCACAAGAAATAGC -3′; Col1a1 F-5′- GCTCCTCTTAGGGGCCACT-3′, R-5′- CCACGTCTCACCATTGGGG-3′. PCR mix contained optimal concentrations of primers, cDNA and SYBR Green PCR Master Mix (Life Technologies, NY, USA). Relative gene expression was normalized to β-actin and represented as fold change as previously described [60].

### 4.6. Western Blot Analysis

Tissue was lysed with RIPA lysis buffer (50 mM Tris, pH 8, 150 mM NaCl, 1% Nonidet P-40, 0.1% SDS, 0.5% sodium deoxycholate), 0.5 mM PMSF and 1× protease inhibitor (10 µL/mL). Cellular debris was removed by centrifugation at 15,000 rpm for 15 min at 4 °C. Protein content of the clarified lysate was determined using bicinchoninic acid (BCA) reagents from Thermo Scientific (Rockford, IL, USA). Isolated proteins were denatured in SDS gel buffer, separated by SDS-PAGE, and immunoblotted using appropriate primary antibodies. Goat anti-rabbit or anti-mouse IRDye 680 (Cat# 926-68071) or IRDye 800 (Cat# 926-32210) secondary antibodies from LiCor were used for detection and quantitation of immunoblots. Membranes were imaged using a LiCor Odyssey scanner, and blots were analyzed by Image Studio 4.0 analytical software (LiCor, Lincoln, NE, USA) as previously described [61,62]. Primary antibodies included PPARγ (Cat#2435), Fatty-Acid Synthase (Cat#3180), SCD1 (Cat#2438S), β-Actin Mouse mAb (Cat#3700S) from Cell Signaling Technologies (Danvers, MA, USA) and β-actin (Cat#PA5-59497) from Invitrogen (Carlsbad, CA, USA).

### 4.7. Liver Tissue Histological Analysis

Paraffin embedded sections were stained with hematoxylin and Eosin (H&E). Briefly, formalin processed tissues were transferred to 70% EtOH and sent to the Texas A&M Veterinary Pathobiology department for processing, embedding and sectioning. After deparaffinization and rehydration, slides containing paraffin sections were stained with hematoxylin and eosin. After mounting and drying, the slides were scanned in a Brightfield microscope at 10× and 40× magnifications for photomicrographs.

### 4.8. Statistics

All results were expressed as mean ± SEMs. One-way and two-way ANOVA with Tukey’s post hoc test were performed as appropriate across all data sets. Additionally, *t*-tests were also performed as needed across specific data sets. The exact *p* value is documented on the graph where two specific groups were compared.

Statistical significance was determined by *p* < 0.05. Statistical analysis was performed using Prism 6.0 (San Diego, CA, USA).

## Data Availability

Exclude this statement as there is not data reported.

## Supplementary Materials

The following are available online at https://www.mdpi.com/1422-0067/22/4/1822/s1, Supplementary Figure S1. Food consumption was measured in LFD, HFD, HFD-HF cages at weeks 0, 6, 12, and 16; Figure S2. High fat and high fat-high fructose diets increase WAT (White Adipose Tissue) and SubQWAT (Subcutaneous Adipose Tissue) weight in male and female mice. WAT and SubQWAT weight of C57BL/6J male (A) and female (B) mice placed on LFD for 20 weeks were measured; Figure S3. mRNA expression of fibrosis regulator in male liver. Expression changes of PAI-1 in male liver among the LFD, HFD, and HFD-HF groups for 20 weeks; Supplementary Table S1. Rodent Diet with 45 kcal% Fat and Modified Version with 17 kcal% Fructose and 17 kcal% Sucrose, and Low-Fat Control Diets.

## Abbreviations

LFD (low-fat diet), HFD (high-fat diet), HFD-HF (high-fat high-fructose diet), PPAR (Peroxisome proliferator-activated receptor), GTT (glucose tolerance test), ITT (insulin tolerance test), FAS (fatty acid synthase), Stearoyl-CoA desaturase-1 (SCD1).
