# Supplementary material for: Effect of Chronic Western Diets on Non-Alcoholic Fatty Liver of Male Mice Modifying the PPAR-γ Pathway via miR-27b-5p Regulation
Source: Int J Mol Sci. 2021 Feb 12;22(4):1822. doi: 10.3390/ijms22041822 (PMC7917964; doi:10.3390/ijms22041822)
Supplement: Supplementary file 1 [file ijms-22-01822-s001.pdf]

Effect of Chronic Western diets on non-alcoholic fatty liver of male mice by modifying the PPAR-γ pathway via miR-27b-5p regulation

Jian Zhang, Catherine A Powell, Matthew K Kay, Ravi Sonkar, Sunitha Meruvu, Mahua Choudhury*
Affiliation: Pharmaceutical Sciences, Texas A & M Health Science Center
* Corresponding Author

Food consumption

Food consumption was calculated by measuring food consumed over a 3 day period (e.g., 4 pm on Friday evening, ending at 4 pm on Monday evening). The weight of diet and feed rack were measured at 0 h and after 72 h (including diet in the bedding). Calculation was performed as follows:

(1) A=Beginning weight (g)-Ending weight (g),

(2) B=A/mice number in the cage= Average food consumption in grams per mouse per day

(3) C=B/3 days=Food consumption

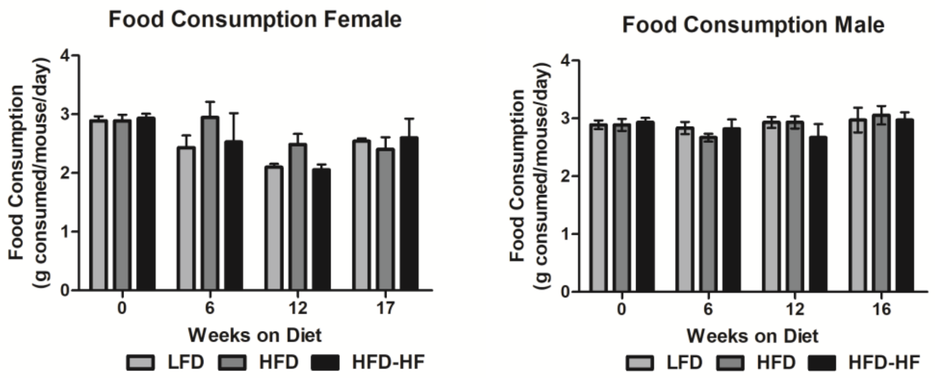

Figure S1 Food consumption was measured in LFD, HFD, HFD-HF cages at weeks 0, 6, 12, and 16. N=4 cages/group, two-way repeated measures ANOVA analysis was performed.

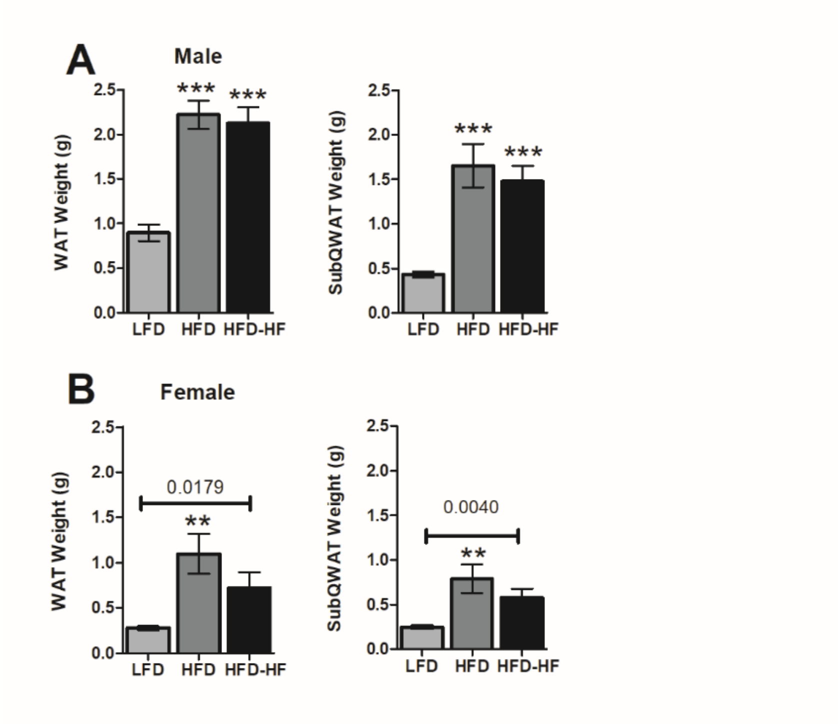

Figure S2 High fat and high fat-high fructose diets increase WAT (White Adipose Tissue) and SubQWAT (Subcutaneous Adipose Tissue) weight in male and female mice. WAT and SubQWAT weight of C57BL/6J male (A) and female (B) mice placed on LFD for 20 weeks were measured. n=10 mice/group, one-way ANOVA, **P<0.01 VS LFD, ***P<0.001 VS LFD, and t-test.

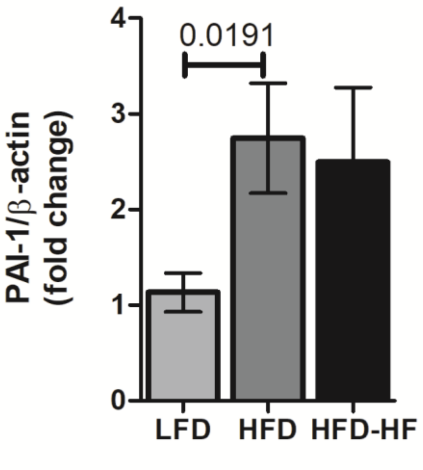

Figure S3 mRNA expression of fibrosis regulator in male liver. Expression changes of PAI-1 in male liver among the LFD, HFD, and HFD-HF groups for 20 weeks. n=7 mice/group, t-test. PAI-1 F-5'- AGAAACGGGACAAACTTCGTC-3', R-5'- GTCTTGCACTGTATAGCCGAG-3'.

Table S1 Rodent Diet with 45 kcal% Fat and Modified Version with 17 kcal% Fructose and 17 kcal% Sucrose, and Low-Fat Control Diets

	HFD		HFD-HF		LFD	
**Product #**	**D12451Y**		**D15041701**		**D12450H**	
	gm%	*kcal%*	gm%	*kcal%*	gm%	*kcal%*
**Protein**	24	*20*	24	*20*	19	*20*
**Carbohydrate**	41	*35*	41	*35*	67	*70*
**Fat**	24	*45*	24	*45*	4	*10*
**Total**		*100*		*100*		*100*
**kcal/gm**	4.7		4.7		3.8	
**Ingredient**	gm	*kcal*	gm	*kcal*	gm	*kcal*
**Casein**	200	*800*	200	*800*	200	*800*
**L-Cystine**	3	*12*	3	*12*	3	*12*
**Corn Starch**	72.8	*291.2*	0	*0*	452.2	*1808.8*
**Maltodextrin 10**	100	*400*	0	*0*	75	*300*
**Sucrose**	172.8	*691.2*	172.8	*691.2*	172.8	*691.2*
**Fructose**	0	*0*	172.8	*691.2*	0	*0*
**Cellulose, BW200**	50	*0*	50	*0*	50	*0*
**Soybean Oil**	25	*225*	25	*225*	25	*225*
**Lard**	177.5	*1597.5*	177.5	*1597.5*	20	*180*
**Mineral Mix S10026**	10	*0*	10	*0*	10	*0*
**DiCalcium Phosphate**	13	*0*	13	*0*	13	*0*
**Calcium Carbonate**	5.5	*0*	5.5	*0*	5.5	*0*
**Potassium Citrate, 1 H2O**	16.5	*0*	16.5	*0*	16.5	*0*
**Vitamin Mix V10001**	10	*40*	10	*40*	10	*40*
**Choline Bitartrate**	2	*0*	2	*0*	2	*0*
**FD&C Yellow Dye #5**	0.05	*0*	0	*0*	*0.04*	*0*
**FD&C Red Dye #40**	0	*0*	0.05	*0*	*0.01*	*0*
**FD&C Blue Dye #1**	0	*0*	0	*0*	0	*0*
**Total**	**858.15**	***4057***	**858.15**	***4057***	**1055.05**	***4057***
